# Protecting Personhood: A Classic Grounded Theory

**DOI:** 10.1177/10497323231190329

**Published:** 2023-09-05

**Authors:** Amélia Didier, Alvita Nathaniel, Helen Scott, Susanne Look, Lazare Benaroyo, Maya Zumstein-Shaha

**Affiliations:** 1 School of Health Sciences (HESAV), 87680 HES-SO University of Applied Sciences and Arts Western Switzerland, Lausanne, Switzerland; 2 Department of Nursing, 5631 West Virginia University, Morgantown, WV, USA; 3 Grounded Theory Institute, Mill Valley, CA, USA; 4 Grounded Theory Institute, Chichester, UK; 5 9231 Vivantes Hospital Group, Berlin, Germany; 6 Interdisciplinary Ethics Center, 27213 University of Lausanne (UNIL), Lausanne, Switzerland; 7 Bern University of Applied Sciences Health, Bern, Switzerland; 8 Department of Nursing, University of Witten/Herdecke, Witten/Herdecke, Germany

**Keywords:** behavior, communication, trust, doctor–patient, nurse–patient, lived experience, health, users’ experiences, healthcare, holistic care, experiences, illness and disease, theory development, methodology

## Abstract

The importance of perceiving and considering patients as healthcare partners has been increasingly promoted. Healthcare systems around the world are now highly interested in patient engagement, participation, collaboration, and partnership. Healthcare professionals are advised that patients, as autonomous beings, should be active in and responsible for a portion of their own care. The study presented here focused on patients’ perceptions of interprofessional collaboration. It was conducted using the classic grounded theory methodology. The theory of protecting personhood emerged as the core concept of hospitalized patients, cared for by interprofessional healthcare teams. This theory encapsulates the process hospitalized patients go through to find balance in their sense of self, oscillating between personhood and patienthood in the unfamiliar hospital environment. The process consists of four stages: the stage of introspection, during which hospitalized patients become aware of their self as a person and as a patient; the stage of preservation, when patients find a balance between the sense of personhood and patienthood; the stage of rupture, wherein patients experience an imbalance between their sense of personhood and patienthood; and the stage of reconciliation, in which personhood is restored. The theory of protecting personhood offers insights into a better understanding of hospitalized patients’ experiences and strategies, revealing the importance of relationships, and the driving force of empowerment. This study is about patients’ perspectives of interprofessional healthcare teams. A grounded theory process allowed the emergence of patients’ concerns and expectations, leading to a substantive theory grounded in the patients’ data.

## Background

Patients’ value as healthcare partners has been recognized and promoted during this last decade. According to a patient-centered model and definition of interprofessional collaboration, patients have the potential to act on each level of care: on a direct level (Gausvik et al., 2015), on an organizational level, and on a policy level of care (Institute for Patient- and Family-Centered Care [IPFCC], 2017; Ocloo & Matthews, 2016). The concepts of patient engagement, partnership, and participation are of high interest in current healthcare systems around the world (IPFCC, 2017; WHO, 2017; Wilcock et al., 2003) and in Switzerland (Swiss Medical Sciences Association, 2020). Patients, as autonomous beings, are encouraged to participate in decision-making to be active in and responsible for their own care and safety in healthcare (Holmström, & Röing, 2010; IPFCC, 2017; Hôpitaux Universitaires Genève [HUG], 2019). The World Health Organization (WHO, 2017) recommends that healthcare professionals include patients as active participants in monitoring their care and improving their healthcare outcomes. In the United Kingdom, patient partnership and engagement were integrated into the National Health Service (NHS) more than two decades ago and form part of the professional standards (WHO, 2013). In Switzerland, interest in person-centeredness and partnership is increasing. In fact, the Swiss Medical Sciences Association (2020) insists on the importance and value of patients as partners in its revised Interprofessional Charter.

Engagement, partnership, collaboration, and patient-centeredness differ in gradation and meaning, which affects the roles assigned to patients. The differences in meaning also influence the definition that professionals or patients assign to the concept of patient inclusion or patient-centeredness in healthcare (Canadian Interprofessional Health Collaborative, 2010; Karazivan et al., 2015; Ocloo & Matthews, 2016) or the way these concepts are implemented in practice (Phillips & Scheffmann-Petersen, 2020). Engagement is defined as a continuum, spanning from consultation through involvement to partnership. Participation means taking part in, for example, the care process or decision-making (Arnetz et al., 2016; Thórarinsdóttir & Kristjánsson, 2014), whereas partnership is the highest level of patient engagement in the process, whether at the level of direct care, organization, or policy (Ocloo & Matthews, 2016).

Similarly, the person-centered and/or patient partnership models encourage patient involvement at micro, meso, and macro levels of the system: in policymaking, in clinical decision-making processes, or in educational programs for healthcare professionals (HUG, 2019; Karazivan et al., 2015; McCormack & McCance, 2016; Thórarinsdóttir & Kristjánsson, 2014). Models of patient- or person-centeredness (McCormack & McCance, 2016; Kitwood, 2011; Langberg, 2019), and respective organizations or institutes such as the “Institute for Patient- and Family-Centered Care,” have emerged in the last decades, promoting partnership with patients and persons and their families to ensure their empowerment in care, research, and education and to improve patient outcomes.

In some contexts, partnership, participation, person-centered care, communication, and collaborative practices are claimed as the standards of care and are encouraged. However, they remain difficult to implement because of patients’ and/or healthcare professionals’ beliefs about the patients’ roles, power issues, relationships between healthcare professionals and patients (Larsson et al., 2007; Phillips & Scheffmann, 2020), and “gaps between policy and practice” (Phillips & Scheffmann-Petersen, 2020, p. 1420; Zoffmann et al., 2008). In addition, neither patients nor healthcare professionals always know how to deal with those standards in practice (Martin & Finn, 2011; Phillips & Scheffmann-Petersen, 2020). Both patients and healthcare professionals need guidance on how to live patient-centeredness (Phillips & Scheffmann-Petersen, 2020) and collaborative practices (Phillips et al., 2015).

However, there are indications that differences exist concerning the respective roles and the reinforcement of patients’ healthcare competencies, such as healthcare literacy and knowledge of healthcare issues. Care of persons with chronic illness, for example, relies on self-management and assessment of symptoms and treatments, as well as on shared decision-making between patients and healthcare professionals (Friesen-Storms et al., 2015; Thórarinsdóttir et al., 2019). Nevertheless, healthcare environments are still strongly influenced by issues like economic levers, which may cement patients’ passive roles. This perceived passive role may further be affected by health literacy. In Switzerland and in other countries, patient literacy remains low (N’Goran et al., 2018), curtailing confidence and the intention to participate in interprofessional discussions and decisions. In such environments, patients await education and healthcare instead of actively requesting them (Crisp, 2012). On the one hand, patient engagement, participation, and collaboration are promoted under these circumstances, but on the other hand, patients do not always feel authorized to act, nor do they know how to act (Phillips & Scheffmann-Petersen, 2020).

Patients’ perspectives and experiences of patient-centeredness and interprofessional collaboration have been studied in some areas such as intensive care (Gausvik et al., 2015) and rehabilitation (Zimmermann et al., 2014), as well as in the community (Giusti et al., 2022; Phillips et al., 2015) and in oncology (Giusti et al., 2022). The flow and coherence of communication among various healthcare providers has often emerged as being problematic and provoking uncertainties as well as negative patient experiences (Gausvik et al., 2015). Interprofessional care provision involves acknowledgment of the various healthcare providers’ backgrounds and education and finding ways to communicate with one another to provide coherent and tailored information to patients (Gausvik et al., 2015).

In order to find ways to overcome these challenges, it is important to determine patients’ experience of interprofessional collaboration and to ascertain the best way for patients to join in their care as part of a collaborative process. To our knowledge, despite a large body of evidence concerning patients’ involvement in care and healthcare communication, little evidence exists regarding patients’ perspectives of their experiences of interprofessional collaboration or of collaborative practices in the hospital environment. Except in decision-making (Gulbrandsen et al., 2016), some evidence exists respecting patients’ readiness to partner or participate in, collaborate on, or actively engage in collaborative processes such as interprofessional collaboration in hospitals. There is also some evidence as to healthcare professionals’ prerequisites for enabling such processes.

This study was part of a larger research project on interprofessional collaboration. The aim of the larger project was to explore the collaborative process between healthcare professionals at a managerial level. The purpose of this portion of the larger project was to examine patients’ perspectives on interprofessional collaboration within multidisciplinary or interprofessional healthcare teams. Thus, the original research question for this study was “what are patients’ perspectives of interprofessional collaboration?” The literature highlights that patients and interprofessional collaboration, participation, or engagement go beyond a question of perspective. It includes patients’ views, experiences, emotional responses to relationships with healthcare professionals, and the human connection between them and the healthcare professionals (Larsson et al., 2007; Thórarinsdóttir & Kristjánsson, 2014).

Therefore, classic grounded theory was chosen as the most appropriate research method for this investigation. After data collection and analysis began, the research question evolved, as is common with inductive grounded theory research. The question was “what is going on with patients when they are cared for by interprofessional teams?”

In addition, grounded theory answers the following questions: What is the main concern of this group of people? How is this main concern continually resolved? Grounded theory was well-suited to this study because its methodology allows for an in-depth understanding of processes, actions, and interactions that participants go through, allowing for a grasp of how they view and experience these processes.

## Methods

### Design

A qualitative study design was selected for this study to allow patients to openly express their concerns during their hospitalization under an interdisciplinary healthcare team. This research was based on the classic grounded theory (GT) research method. As such, participants’ genuine concerns, strategies, actions, and interactions were elicited step-by-step based on the classic GT process. The classic GT research method requires the analyst to remain close to the data and to limit interpretation to determine the patterns in the data. Conceptualization was achieved through the GT process of constant comparison of coded data, from which concepts emerged. Further relationships between concepts were identified through a theoretical coding process.

### Participants/Sampling Methods

This study was conducted in two adult surgery departments (neurosurgery and ear, nose, and throat surgery) in a university hospital in the German-speaking part of Switzerland between July 2016 and June 2017. The sample consisted of 32 adult patients, comprising 15 women and 17 men, with a mean age of 54 years. The majority of the patients were Swiss; only three patients originated from Southern or Eastern Europe, and two patients were from Western Europe. The patients’ levels of education varied between the secondary level, that is, compulsory and apprenticeship (*n* = 25), and the tertiary level (*n* = 7). Three of the patients in the secondary-level category had businesses of their own. The patients were undergoing elective (*n* = 17) as well as emergency (*n* = 15) procedures. The average length of stay was 5.2 days, with a minimum stay of one day and a maximum stay of 12 days. The length of stay for each patient tended to be longer in the neurosurgical service than in the ear, nose, and throat service.

Participants were hospitalized for at least one day and cared for by interdisciplinary healthcare teams which included physicians, nurses, nursing assistants, physiotherapists, occupational therapists, dieticians, and chaplains, among others.

### Data Collection and Analysis

Data was collected through face-to-face interviews. These exchanges commenced with a general question about patients’ experiences with the service: “How was your experience in the interprofessional care environment?” Follow-up questions to probe and clarify issues raised by participants allowed for their perspectives to be more thoroughly explored. The different steps of classic GT were followed, including simultaneous data collection and analysis, interview transcription, substantive coding (open and selective coding), constant comparison, theoretical sampling, memoing, and sorting. These steps facilitated the emergence of participants’ main concerns and the core category of this substantive theory. The core category is of central importance in GT because it “accounts for most of the variation in the pattern of the participants’ behavior” (Glaser, 1978, p. 93). As such, the core category constitutes the fundamental pattern of a phenomenon; it has explanatory power, and all other concepts are linked to it (Glaser, 1978).

### Rigor of the Study

A classic GT is considered sound when it is relevant, it works, it fits to the data, and it is modifiable (Glaser, 1978). As suggested by both Glaser and Charmaz, constant comparison and memoing assured fit with the data (Charmaz, 2014; Glaser, 1978). Also, as suggested by Glaser (1978), Charmaz (2014), and Birks and Mills (2022), discussions and debates between the researcher and the supervisor, and subsequently with patients and healthcare professionals, confirmed that the words and language in the theory reflected the participants’ experiences and that emergent categories were grounded in the data. The need to translate the incidents and concepts from German to English posed a challenge that was overcome through in-depth discussions and debates between the researcher and the supervisor, who is a native German and English speaker, and subsequently with the GT mentor, who is a native English speaker. Participant quotations are included in the following sections to illustrate a basis for the construction of the categories and provide context.

### Ethical Considerations

In Switzerland, the processing of personal and sensitive data is protected by the Federal Data Protection Act and the Cantonal Data Protection Act. The study protocol was submitted to the local cantonal ethics committee and to the institutional pediatric ethics committee. The data presented did not fall under the Human Research Legislation (Swiss Confederation, 2014) as the data collected did not include health-related data specifically. However, each participant received written information on the study, had time for reflection, and returned a signed consent form. All data was deidentified, and confidentiality was guaranteed to study participants.

## Results: The Theory of *Protecting Personhood*

Theories include inherent assumptions, conditions, and some level of context. This theory posits that (a) hospitals are neither a natural nor a familiar environment for people who have rarely or never had health issues and that (b) hospitals’ structural functioning is unknown to healthy people who have no interaction with the healthcare system. Hospitalization can be a hugely disruptive life event. Becoming a healthcare patient and learning to interact with healthcare professionals are adaptive and sometimes challenging processes. Once admitted to hospitals, patients enter a dynamic process and adopt strategies, attitudes, and behaviors to secure the care they want to receive. This substantive theory explains how patients activate processes to protect and maximize their personhood to receive optimal care.

**Figure 1.**
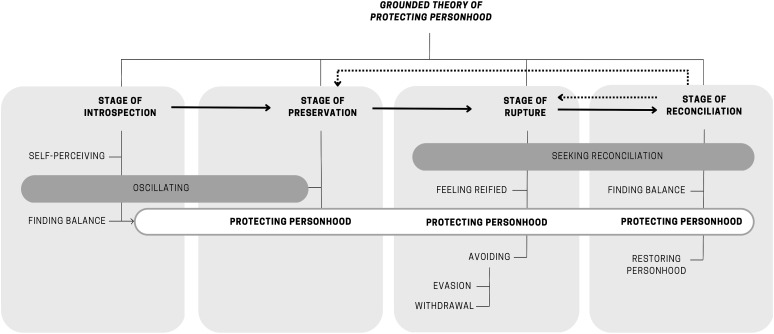
Grounded theory of protecting personhood.

The grounded theory of *protecting personhood* thus encapsulates the process that hospitalized individuals go through to find balance in their sense of self, oscillating between personhood and patienthood in unfamiliar hospital environments. The process consists of four stages: introspection (when hospitalized individuals become aware of their self as a person and as a patient); preservation (when individuals find a balance between the sense of personhood and patienthood and personhood is protected); rupture (imbalance between the senses of personhood and patienthood, wrecked personhood); and reconciliation (when personhood is restored), as illustrated in Figure 1.

Originally written and conceptualized in German, the process of protecting personhood was encapsulated in a German term, *Aufgehobenheit*, with no absolute English equivalent. The term used by the patients when they were receiving safe and protective care was feeling “aufgehoben.” Aufgehoben started to emerge as an umbrella term in the memos and field notes, with the power of summarizing an optimal care moment during the interactions and relationships between the patients and the healthcare professionals. Feeling “aufgehoben” during care moments with healthcare professionals had the power to transform any encounter with the healthcare professionals into a positive, special, and dynamic experience. The adjective “aufgehoben” was transformed into the noun “Aufgehobenheit” to stress its potential as a process and core concept. Constant comparison ensured the concept’s fit with the patients’ data.

After careful consideration, protecting personhood was chosen as the nearest English term to represent the concept of Aufgehobenheit. Protecting personhood was identified through the GT analytical procedures of selectively coding the field notes, conducting constant comparison, and writing memos. Analysis revealed that patients not only seek to receive good care and feel safe and protected but are also concerned with their relationships and interactions with healthcare professionals, how those interactions unfold, and how to provoke a change when needed. Protecting personhood was retained as the core category because it had the most explanatory power in the theory and explained how participants continually resolved their main concerns.

### Stage 1: Introspection

The first stage of the theory of protecting personhood is *introspection.* This stage highlights the process the hospitalized individuals pass through as they perceive a change in their condition: they notice that they move from the person they are to being the patient. The person becomes aware of this change due to the diagnosis and/or future hospitalization. Awareness of this change triggers the process of introspection, which in turn leads to the concept of self-perception. It is a kind of transition in the mind. Every time the patients talk about entering the hospital environment, they begin a phase of introspection on their conditions. The following comment made by Olivia (a patient) illustrates one patient’s stage of introspection, which allowed for the emergence of the concept of self-perceiving: “… when you are at home, and you know you need to be hospitalized, you feel up and down. And as soon as you are here [hospital], you close the door behind you, you wear your [patient] gown, you are like … not yourself anymore. You are in others’ hands …”

Introspection includes the properties of self-perceiving, oscillating, and finding balance.

#### Self-Perceiving

Prior to the first hospitalization, and before any encounter with healthcare professionals, persons are more or less healthy, are more or less autonomous, have their own habits and ways of being, are part of a family and of specific organizations, retain their sense of dignity, and hold their own opinions. At hospital admission, patients enter an unfamiliar environment and put their lives into the hands of unfamiliar persons, healthcare professionals. When entering the unfamiliarity of the hospital, the individuals undergo changes which can provoke a variation in their perception of themselves. Individuals start perceiving themselves as patients: they perceive that they are the same person with a specific condition, a medical condition. The awareness of themselves with a medical condition provokes introspection with a slight change in their perception of themselves and their identity as a person. The concepts of personhood and patienthood as a state start to emerge more or less consciously. Still, the state of personhood is not to be considered the opposite of patienthood. Rather, personhood and patienthood constitute two dimensions of the hospitalized individual, which are strongly intertwined. In this stage of introspection, however, the individuals are in a state of oscillation. They are both the one and the other.

#### Oscillating and Finding Balance

The hospitalized individuals are oscillating between the state of person and patient; thus, they are constantly driven by their desire and need to remain connected to their personhood.

This process is conceptualized as “oscillating” because of the movement the patients undergo from the state of person to the state of the patient, and back. The concept of patient does not exclude the concept of person. Both must cohabitate in harmony. The questions triggered during this process are “Am I considered a person? Am I feeling myself to be a person?” If the interactions and relationships with healthcare professionals are optimal, the response in that process will be “I am a patient right now, but I feel like a person.” Once that balance is found, the hospitalized individuals enter into a stage of preservation. For example, Olivia (a patient) who perceived the change between her personhood before entering the hospital and the transition to patient-condition stated, “I need to feel that I am considered as a patient. Yes. To know that I matter to them [the health professionals], that there is a person.”

Another patient, Jürgen, helps to understand the emergence of the need to be considered as a person: “[…] and not just having the nurse asking only about my pain and leave.” Jürgen maintains the need to be listened to, to be seen, and to be understood in full as a person. He needs to find a balance between his condition as a patient and his personhood.

As long as the state of “person” is not obtained, the patient is oscillating in between.

The consequence of oscillation is finding a balance. This means being a patient and still feeling like a person, that is, how persons protect their personhood and remain connected to their own sense of personhood. Moments of care may reinforce or jeopardize the process of oscillation. The interactions and relationships between individuals and healthcare professionals will affect individuals’ self-perception.

#### Stage 2: Preservation

The second stage of protecting personhood is *preservation.* Preservation concerns the process patients undergo in an unfamiliar environment. In these circumstances, the patients do not know much about the environment, the actors (i.e., the healthcare professionals), or the types of interactions patients will face or witness. The patients will do their best, or transmit signs, to indicate to healthcare professionals ways to help preserve the feeling of personhood, while being in a potentially “debilitating” environment. This stage is tenuous because it can easily vary according to patients’ expectations and experiences of care, their interactions and relationships with healthcare professionals, and the context and atmosphere of the care environment.

#### Protecting Personhood

During the process of protecting personhood, the aim of the hospitalized individuals is to remain connected to their personhood and continue to feel themselves to be a person no matter the circumstances of care, relationships, or interactions. At this time, patients aim to limit uncertainty and discomfort due to the environment and/or relationships they may feel during care. Protecting personhood is a positive feeling that must be echoed by the behavior of healthcare professionals. Both healthcare professionals and patients must make efforts to protecting personhood. Care moments are experienced as “protective” of personhood when individuals seek a sense of consideration, feel respected in their dignity and autonomy, and feel heard and understood. They need to feel they are in safe hands and provided with consistent information when they ask for it. The question at this stage might be: “Am I heard about the issues I address? Is the healthcare professional comforting, caring? Do the healthcare professionals consider me a person?”

Healthcare professionals’ behavior and attitudes and their interactions and relationships with patients have the power to generate feelings and atmospheres of safety, respect, consideration, and dignity. The quotes below help to define the concept of protecting personhood.

Tina said, “It has something to do with the state of mind, the feelings, the presence … You are given something, you are not just a number. They talk to you, they call you by your name, and they even remember what you said the next morning ...” Susanna remarked, “Well, this time I think, I was taken seriously. I had privacy; I was allowed to shower by myself, and so on. Well, the first time I was washed in the recovery ward, but even that was really done in such a way that I felt respected in my privacy. It was not like that before [previous hospital stay]. I was just put in the shower and scrubbed.”

Thus, patients recognize that healthcare professionals’ behavior and attitudes either generate or inhibit feelings of safety, respect, consideration, and dignity.

### Stage 3: Rupture

The stage of *rupture* is a consequence of non-preservation of personhood. Rupture includes the properties of *feeling reified* and *avoiding.* Early in this stage, individuals seek to protect their personhood in relationships and interactions with healthcare professionals. A rupture in the process of protecting personhood occurs when they fail to do so. The balance reached through oscillation and maintained in the preservation stage is wrecked because healthcare procedures and/or the behavior of healthcare professionals do not meet patients’ expectations and needs to be provided with consistent information, and with safe and protective care. In this case, the individuals as patients feel disconnected from their personhood. The individuals no longer perceive themselves as respected and considered as persons in their patient-condition.

The conditions leading to the disconnection and rupture in the process of protecting personhood, with individuals’ distortion of self, are a perception of negative and suboptimal care moments and interactions. The less the patients feel protected in their personhood, the closer they come to feeling disconnected and reified.

When healthcare professionals do not engage in *protecting-personhood-*generating behaviors that provide or restore a sense of safety and protection, the distortion of patients’ personhood can continue, reinforcing anxiety and mistrust toward the care environment. As a result, the person feels helpless.

#### Feeling Reified

*Feeling reified* is an important concept in the rupture stage. When both healthcare professionals and patients fail to protect personhood, to maintain the balance between personhood and patienthood, patients start feeling dehumanized and their sense of patienthood dominates negatively, with a focus on disease, on their dependence on healthcare professionals, and on their potential limitations. Feeling reified occurs when they start perceiving themselves as objects. This process happens when patients experience a distortion of their self-perception, caused by a profound feeling of being disrespected or discarded. They feel like an object, a number, an animal. The following incident, for example, is one of those which shaped the concept of reification. Justin said, “On Friday, I was waiting to leave the hospital, and the nurse came in and told me: ‘We need your bed. We are waiting …’ … And I replied that I was not aware that I could leave. It had only been suggested that morning. She countered saying, ‘Yes, you are leaving, your bed is already assigned to someone else’ … For a moment, I felt that I was expendable [patient laughs] …”

Thus, Justin felt disrespected and discarded.

The process of reification is not irreversible. Patients are still striving to activate the process of protecting personhood. However, ongoing or non-resolved disruptive verbal or procedural interactions lead to changes in individuals’ attitudes and behaviors toward the healthcare environment and professionals. The individuals start mistrusting healthcare professionals.

#### Avoiding

An ongoing feeling of reification can lead to an avoidance strategy. This strategy encompasses evasion or withdrawal.

Evasion leads to the person’s decision to leave or not return to the environment where the rupture occurred. Long-term evasion may not be possible. In some cases, alternative options to obtain treatment at a distance from the source of rupture and reification may not be feasible for patients. In such circumstances, when they must return to the source of rupture, patients may engage in care moments with an attitude of withdrawal, withdrawing from relationships with healthcare professionals.

In these cases, patients no longer make any requests, as they have lost their trust in healthcare professionals. Patients may then act on their own. These are two examples of incidents that led to the concept of withdrawal. Ingrid explained, “Well, the level of trust has dropped, because I had no answers. That is why I decided not to go to those physicians anymore.” Another patient, Tina, said, “[I want] nothing [to do] with her [nurse] anymore. I did not say a thing. […] I thought, what for? It is no use; I will be home again soon.”

### Stage 4: Reconciliation

The key strategy to reconnect with personhood lies in activating the process of protecting personhood during care moments. The process of protecting personhood enables patients to adapt to their environments and patients’ condition without losing their sense of being a person. Despite the patients’ strategies, rupture may occur. However, the patients are constantly on the lookout for ways to adapt and remain connected to their personhood. They strive for the sense of personhood, trying to move away from the perspective of themselves as patient-object.

#### Seeking Reconciliation

The rupture and *reconciliation* stages are closely intertwined. Patients do not wait for rupture to be complete to activate the reconciliation process. In the early stage of rupture, the process of protecting personhood is still triggered, aiming to reconcile very promptly with their personhood, before switching to avoidance. When the process of protecting personhood is compromised, patients seek to repair the moment by reactivating optimal care and thus generate the process of protecting personhood. Seeking reconciliation means that patients do try to restore their self-perceptions of themselves as persons.

The reconciliation stage is obvious when the individuals start asking numerous questions, taking measures, and making suggestions to the healthcare professionals. These actions may be perceived as complaints, but they are alerts. Patients do not intend to complain, nor are they searching for errors or inconsistencies in the care they receive. Quite the contrary, in the process of protecting personhood to find a new balance, patients aim to counterbalance the rupture. No matter the reason for rupture and reification, patients do not necessarily blame the healthcare professionals. Patients are conscious of the organizational aspects behind healthcare professionals’ attitudes and behaviors which lead to rupture. They understand that disruption in the care moment and environment, in interactions, or in relationships with healthcare professionals is not always due to a lack of respect or consideration of their person. Patients perceive and observe the various influences on healthcare professionals’ attitudes and behaviors, such as time constraints, work overload, or lack of role clarity. The adopted strategies are intended to provoke a change in the healthcare professionals’ behaviors and procedures. To achieve reconciliation, patients ask, react, or complain. Patients seek to be heard, to be reassured, or to feel safe. Some patients may be assertive. They have developed ways to obtain the information they need. For example, Gert explains: “[…] And then I also spoke up to the doctor: The first antibiotic had not been ordered correctly in my opinion, it had not been of any use. And then he told me: Yes, it was actually not suitable, the antibiotic. Do you understand? That’s what I mean when I refer to my critical attitude.”

Others explain how they get to grips with disruptive situations, such as Olivia: “[…] I have to get rid of such things [negative experiences] … and it has been cleared up immediately … yes … I do not carry that all along and hold a grudge … it is best for everyone …”

Patients’ reactions are variable: the following incident, experienced by Esther, helps to give an idea of other ways in which patients try to trigger the reconciliation process, to elicit a reaction from healthcare professionals and thus the lever for reconciliation: “[…] And I am very aware of that [other priorities, emergencies that healthcare professionals need to address], maybe others [other patients] are not, and they start yelling. I do not do that. I was sitting here and crying on Wednesday.”

For the process of reconciliation to be achieved, patients need to be reassured and sense that they can feel safe, protected, listened to, and considered again. During that process, a good way to allow reconciliation is to show willingness to listen, to be caring and to integrate the patient into the care coordination, and to discuss and provide consistent information on care procedures and results in an understandable way, quickly and in time. If the reconciliation stage is achieved, the person finds balance between the state of personhood and patienthood and can return to preservation, as Gert did, for example: “I was relieved because I told him, and he did not deny it. He admitted diplomatically that it [the treatment] was not adapted.” Protecting personhood is ensured, but it remains a dynamic and mutual process.

Reconciliation can occur at different times, in different spaces, or in other interactions with other healthcare professionals. Reconciliation can be delayed and occur at another moment entirely. A previous disruption can be repaired through a protective attitude/behavior/atmosphere in a new but corresponding environment with different healthcare professionals, even long after the initial rupture. Such reconciliation is illustrated by Tina, who has had a bad experience in the past but has reconciled with the care environment and her sense of personhood: “… nowadays, the person is surely more central […] now I am here, and everything is perfect!”

With every new care moment comes a new opportunity to activate the process of protecting personhood and achieving reconciliation between personhood and patienthood. When healthcare professionals engage in the process, they respond with their behaviors to patients’ intentions to restore personhood and optimal care. Such moments are like turning on a switch, as illustrated by Tina.

## Discussion

Substantive grounded theories are explanatory, yet modifiable as new information is gained and extant literature is explored. The following discussion positions the contribution of the theory of protecting personhood in relation to extant literature, offers implications for practice, and suggests avenues of possible further research.

### Integration with Extant Literature

This GT supports the preexisting knowledge that patients need to feel confident and empowered within the healthcare system. They need to experience a humanized care context (Larsson, 2007; Thórarinsdóttir & Kristjánsson, 2014). Our findings fit into these previous studies’ results, but also point out the fragile dynamics of the care process. Patient participation is also influenced by internal factors such as the patients’ own views of participation and emotional responses concerning the relationship between patients and healthcare professionals (Larsson et al., 2007). Thus, the aim of the literature review was to deepen our understanding of and expand the core concept (Glaser, 2012). The substantive theory of protecting personhood explains what matters to hospitalized patients and how patients resolve their main concerns of securing optimal care and preserving their personhood within an interdisciplinary healthcare team. This theory also highlights that patients’ initial concerns are less about interprofessional collaboration itself than about the importance of their relationships with healthcare professionals and the interactions experienced during care moments; this, in turn, influences their attitudes and behavior toward their interdisciplinary healthcare teams. The relationships and interactions between healthcare professionals and patients constitute a key factor in this theory and can drive patients’ experiences in a positive direction or its polar opposite, depending on how the process of protecting personhood evolves. Overall, this theory supports previous findings on the importance of relationships in nursing and healthcare (Kitson et al., 2021; Kitwood, 2011; Peplau, 1992; Phillips & Scheffmann-Petersen, 2020; Thórarinsdóttir et al., 2019; Thórarinsdóttir & Kristjánsson, 2014; Watson, 2018).

Returning to the original German concept of Aufgehobenheit, which encapsulates the process of protecting personhood*,* it was necessary to review the concept in the German literature before reviewing the larger healthcare literature. Aufgehobenheit *is defined* as a theoretical anthropological term which refers to a person’s inner state, a condition of “being” (*das Sein)* (Knapp, 1988). The concept of Aufgehobenheit can be found in the writings of a German psychologist, Gunthram Knapp (1988). Individuals live in the world alongside other human beings; indeed, their interactions with others play a significant role in their own life experiences. The interdependence between the person and others is developed in the early mother–child relationship (called *Primärbeziehung*). In this early mother–child relationship, Aufgehobenheit is a psychoanalytic term used to refer to the person’s developing response to unfamiliar and stressful life moments (Knapp, 1988). Aufgehobenheit, revealed as the process of protecting personhood by the participants of this study, is a feeling, an inner state, and a response developed by contact with other human beings, the healthcare professionals in the healthcare environment during the first interactions. These first interactions will shape future, unfamiliar, and/or stressful experiences such as hospitalization.

Aufgehobenheit has also been refined through the lens of existing concepts in the German healthcare literature. Verres (1999), a German physician and psychologist, has stressed a key concept close to the term of Aufgehobenheit intended for patients with cancer to attain a state of well-being: the concept of *Aufgehobensein* that refers to a feeling of protection, safety, and care. Through Aufgehobensein, patients feel recognized as persons and can accept their condition and/or recover more rapidly (Verres, 1999). In the international healthcare and nursing literature, the nature and importance of relationships during the care moments have been captured in humanistic theories of caring (Watson, 2018), interpersonal relationships (Peplau, 1992), and person-centered care and frameworks (Kitson, 2018; Kitwood, 2011; McCormack & McCance, 2016; Phillips & Scheffmann-Petersen, 2020). The relationship has the power to influence patients’ experiences (Kitson, 2018; Kitwood, 2011; Peplau, 1992; Phillips & Scheffmann-Petersen, 2020; Paterson, 1988). The importance of care relationships, particularly the nurse–patient relationship, has been described as essential in previous GT studies (Cheraghi et al., 2017; Larsson et al., 2007) and is supported by organizations such as the Intstitute for Patient-and Family-Centered Care (IPFCC) (2017), the National Health Service (Wilcock et al., 2003), and the Beryl Institute (Wolf, 2018). For Kitson (2018), the relationship with patients constitutes one of the “bedrocks” of nursing care.

However, neither the relationship nor the process leading to person-centered care is a state. They are not straightforward processes (Gulbrandsen et al., 2016). There are issues of empowerment versus power relations (Calvès, 2009; Gulbrandsen et al., 2016; Phillips & Scheffmann-Petersen, 2020). The focus on the relationship allows for the recognition of the person in the patient (Berntsen et al., 2022; Langberg et al., 2019). The rupture that follows the failure of patients’ strategies to protect their personhood reveals a latent, well-known problem in the current healthcare system, that is, the potential dehumanization of the person, the patient, and the care environment (Fasanelli et al., 2017; Verres, 1999), and the standardization, bracketing, and, ultimately, loss of personhood (Berntsen et al., 2022). According to the theory and the healthcare literature, the person should come first (Kitwood, 2011). However, the preservation of patients as persons, as human beings, can be challenged by factors related to the evolution of the care system and the care environment, leading to a process of dehumanization. The patients did not express the term dehumanization, but they have felt themselves reified and their perception of their personhood distorted.

This substantive theory offers the potential of a lever capable of reversing the process of dehumanization caused by a rupture in the relationship and in the patient’s perception of themselves as persons. However, there is also a need to recognize the potential power relations between healthcare professionals and patients. Phillips and Scheffmann-Petersen (2020) have suggested a mutual and collaborative reflexivity to allow collaborative engagement between patients and healthcare professionals.

Only in this way can reification or dehumanization be repaired. A positive cycle can be created to reduce mistrust, enhance humanized self-perception, and positively influence patients. The higher the level of protecting personhood, the greater the feeling of optimal care, of humanized care.

The innovative aspect of this current substantive theory is, however, that patients activate the process of protecting personhood. During hospitalization, patients do not passively endure the absence of protecting personhood or wait for that process to happen. Contrary to the findings of Oxelmark et al. (2018), who found that patients become more passive in specific conditions, for example, in cases of nurses’ work overloads, this theory shows that patients are always active, no matter the environment or healthcare professionals’ approaches. The patients have empowered themselves to ensure and restore their personhood. Interestingly, some authors have traced the concept of empowerment back to “Freire’s pedagogy of the oppressed” (Calvès, 2009; Holmström & Röing, 2010), from a societal movement not circumscribed in healthcare policy. Passivity may be a strategy, a reaction for alerting healthcare professionals that the environment and/or relationships are disrupted. The other strategies may be visible in the patients’ complaints (Scott & Grant, 2018).

As protecting personhood illustrates, humanization is the mandate of each and every healthcare professional, not just nurses or physicians. In that sense, it is an interprofessional mandate. Medical academics who previously have questioned the concept of establishing therapeutic relationships are coming to recognize its importance (Thibault, 2019). They stress the need to integrate models of humanization into patient care (Thibault, 2019) because healthcare should not only be driven by biopolitical values. Patients are persons not only because of their interactions with others who recognize and respect their personhood (Kitwood, 2011) but also because of their agency and their autonomy (Gulbrandsen et al., 2016). Respecting patients’ autonomy and agency is also a relational process (Gulbrandsen et al., 2016) in which healthcare professionals need to recognize the power relationships inherent in their positions. Brentsen et al. (2022) have stated that depersonalization occurs due to four factors: confusing the patient’s role with the person’s identity, de-individuation, dissimilarity, and denial of agency. They further explain that patients whose agency is reduced are less able to assert themselves. The first step to patient participation is human connection (Thórarinsdóttir & Kristjánsson, 2014). The theory of protecting personhood confirms the importance of human connection and relationship and shows how patients manage to create and maintain it with the collaboration of healthcare professionals.

### Implications for Practice

This substantive theory has pragmatic and important implications for nursing and any healthcare professionals’ practice. Healthcare professionals need to be aware of patients’ expectations and experiences. Future interventions should focus on healthcare professionals’ readiness to empower patients and ways to achieve that empowerment by learning how to share power with the patients. In this study, on the contrary, the patients have empowered themselves to remain persons in an environment in which their personhood was challenged. In a person-centered approach, considering the patient and the healthcare professionals as persons is important because the values, the beliefs, and the reflexivity of the healthcare professionals may influence the ways they interact with the patients they care for and the ways they integrate or empower the patients (Langberg et al., 2019; McCormack & McCance, 2016; Phillips & Scheffmann-Petersen, 2020). The patients need to be reassured and sense that they can feel safe, protected, and considered again. Healthcare professionals may do so by showing their willingness to listen and be caring, integrating patients into care coordination, and providing consistent information on and discussing care procedures and results in an understandable way.

Systematically integrating these aspects into daily practice can help patients protect and restore their personhood, feel human connection, and avoid feeling reified no matter the environment and healthcare professionals’ workloads.

### Implications for Future Research

Considering the implications for practice, future interventional studies should focus on the healthcare professionals’ own sense of protecting personhood. Future research should identify (a) how healthcare professionals develop that sense of protecting personhood for themselves and for their patients and facilities; (b) what prevents them from doing so; (c) which barriers prevent them or facilitators from promoting protective and respectful care; and (d) which are the indicators for implementing interprofessional-based practices that develop professional healthcare attitudes and behaviors to support protective care, regardless of environment and workload.

## Conclusion

This theory is important because it is grounded in patients’ experiences. The current healthcare system is sensitive to patient-centeredness, collaboration, and partnership. However, the stakeholders in the healthcare system need to be aware of existing power relations and the importance of relationships in guiding healthcare professionals to meet patients’ expectations and needs for agency (Gulbrandsen et al., 2016). The patients in this study have described how they function and what they long for in the middle of an unfamiliar environment with multiple healthcare professionals. Interprofessional collaboration did not emerge as a core concept for patients. Despite this, seeking and activating the process of protecting personhood has major implications in a person-centered collaborative process. As suggested by Larsson et al. (2007), participation is influenced not only by external factors related to institutions or healthcare professionals but also by internal factors such as the patients’ own views of participation as well as emotional responses to the relationship between them and healthcare professionals. This theory of protecting personhood stresses the importance of the relationship (Kitson, 2018; Phillips & Scheffmann-Petersen, 2020) and the human connection with the person and may guide interprofessional healthcare teams to identify and understand patients’ strategies.

To involve patients as partners in healthcare teams, we need first to be aware of and understand the patients’ strategies and focus on their expectations. The recognition that patients strive to protect their health as well as their personhood can make it more likely that healthcare professionals will empower patients to join in interprofessional discussions and decision-making processes.
